# Evaluations of training programs to improve human resource capacity for HIV, malaria, and TB control: a systematic scoping review of methods applied and outcomes assessed

**DOI:** 10.1186/s41182-017-0056-7

**Published:** 2017-07-01

**Authors:** Shishi Wu, Imara Roychowdhury, Mishal Khan

**Affiliations:** 1 Saw Swee Hock School of Public Health, National University of Singapore, 12 Science Drive 2 #10-01, Singapore, 117549 Singapore; 2 Communicable Diseases Policy Research Group, London School of Hygiene and Tropical Medicine, Keppel St, London, WC1E 7HT United Kingdom

**Keywords:** Scoping review, Training evaluation, Evaluation methods, Tuberculosis, HIV, Malaria

## Abstract

**Background:**

Owing to the global health workforce crisis, more funding has been invested in strengthening human resources for health, particularly for HIV, tuberculosis, and malaria control; however, little is known about how these investments in training are evaluated. This paper examines how frequently HIV, malaria, and TB healthcare provider training programs have been scientifically evaluated, synthesizes information on the methods and outcome indicators used, and identifies evidence gaps for future evaluations to address.

**Methods:**

We conducted a systematic scoping review of publications evaluating postgraduate training programs, including in-service training programs, for HIV, tuberculosis, and malaria healthcare providers between 2000 and 2016. Using broad inclusion criteria, we searched three electronic databases and additional gray literature sources. After independent screening by two authors, data about the year, location, methodology, and outcomes assessed was extracted from eligible training program evaluation studies. Training outcomes evaluated were categorized into four levels (reaction, learning, behavior, and results) based on the Kirkpatrick model.

**Findings:**

Of 1473 unique publications identified, 87 were eligible for inclusion in the analysis. The number of published articles increased after 2006, with most (*n* = 57, 66%) conducted in African countries. The majority of training evaluations (*n* = 44, 51%) were based on HIV with fewer studies focused on malaria (*n* = 28, 32%) and TB (*n* = 23, 26%) related training. We found that quantitative survey of trainees was the most commonly used evaluation method (*n* = 29, 33%) and the most commonly assessed outcomes were knowledge acquisition (learning) of trainees (*n* = 44, 51%) and organizational impacts of the training programs (38, 44%). Behavior change and trainees’ reaction to the training were evaluated less frequently and using less robust methods; costs of training were also rarely assessed.

**Conclusions:**

Our study found that a limited number of robust evaluations had been conducted since 2000, even though the number of training programs has increased over this period to address the human resource shortage for HIV, malaria, and TB control. Specifically, we identified a lack evaluation studies on TB- and malaria-related healthcare provider training and very few studies assessing behavior change of trainees or costs of training. Developing frameworks and standardized evaluation methods may facilitate strengthening of the evidence base to inform policies on and investments in training programs.

## Background

It is becoming increasingly evident that strong human resources for health (HRH) are essential to improve global health, with recent studies showing that health outcomes are strongly correlated with the quality and density of healthcare providers (HCPs) [1, 2]. Despite remarkable increases in financial support to disease-specific prevention and control programs [3, 4], inadequate HRH is still a major impediment in low- and middle-income countries (LMICs), where diseases such as HIV, malaria, and tuberculosis (TB) cause substantial mortality, morbidity, and negative economic impact [1, 5, 6]. In addition to a shortage in the number of HCPs in LMICs [7], lack of training to improve capacity of staff at different service levels, inadequate geographical distribution within countries, dissatisfaction with remuneration, and low motivation along with poor staff retention contribute to the inconsistent and inadequate quality of services provided by HCPs [8]. As a result of the global health workforce crisis, more funding has been invested in strengthening HRH since 2000. Within HIV, malaria, and TB control programs, training of HCPs has been an area of focus [9]. Between 2002 and 2010, the Global Fund to Fight AIDS, TB and Malaria (the Global Fund)—the largest non-governmental funder of human resources—invested US$1.3 billion for human resource development activities, and it is estimated that more than half of this budget was invested in disease-focused training activities [9]. As a result of increasing attention and investment in strengthening HRH in HIV, malaria and TB control programs, in 2014, the Global Fund provided 16 million person-episodes of training for HCPs, which was a tenfold increase compared to the number trained in 2005 [10].

Along with this investment comes a need for evaluations to provide information for international funders and national program managers to determine if a program should continue, improve, end, or scale up, in order to ensure that resources are allocated effectively and efficiently [11]. However, we found no studies that systematically reviewed existing literature on evaluations of HCP training programs. Furthermore, there is no consensus on best practice in terms of evaluation methods applied and outcome indicators assessed; therefore, summarizing existing literature on evaluations of HCP training is essential.

Among all the frameworks or conceptual models developed to guide conduct of training evaluations, the first and most commonly referenced framework to date is the Kirkpatrick model [12–15]. The Kirkpatrick model has been used in the design of training evaluations in business and industry in the 1960s. It forms the basis of various theories in training evaluation and has had a profound impact on other evaluation models developed subsequently [13, 16–18]. The Kirkpatrick model identifies four levels of training outcomes that can be evaluated: reaction, learning, behavior, and results [19]. The reaction level assesses how well trainees appreciated a particular training program. In practice, evaluators measure trainees’ affective response to the quality and the relevance of the training program when assessing reaction [12]. The learning level assesses how well trainees have acquired intended knowledge, skills, or attitudes based on participation in the learning event. It is usually measured in the form of tests [13]. The behavior level addresses the extent to which knowledge and skills gained in training are applied on the job. Lastly, for the results level, evaluators try to capture the impact that training has had at an organizational level; this includes changes in health outcomes [12].

In light of the growing focus on and investment in improving human resource capacity for HIV, malaria, and TB control and the need for evaluations of these investments, we conducted a systematic review to investigate how frequently HIV, malaria, and TB HCP training programs have been scientifically evaluated, synthesize information on the methods and outcome indicators used, and identify areas for improvement in current training evaluation approaches.

## Methods

This review was based on the systematic scoping review methodological framework designed by Arksey and O’Malley [20]. The following key steps were included when we conducted the review: (1) identifying the research question, (2) identifying relevant studies, (3) study selection, (4) charting the data, and (5) collating, summarizing, and reporting the results.

### Stage 1: identifying research question

The population for this review was HCPs delivering health services related to HIV, TB, or malaria. We included doctors, nurses, healthcare workers, lay health workers, traditional health practitioners, and laboratory technicians in our definition of HCPs. Teachers and other professionals delivering health services outside their routine work were not considered HCPs. The intervention of interest was any training or capacity building activity related to health service delivery. As the purpose of this study is to identify the methods and outcomes used for training evaluations, the study design and outcomes of the included studies were left intentionally broad, and a meta-analysis was not appropriate at this stage.

### Stage 2: identifying relevant studies

We conducted a search on articles published after January 1, 2000, in three electronic databases on April 28, 2016: PubMed, Embase, and Cochrane Library. In addition, we searched for relevant gray literature in Google Scholar (first 100 citations) and on six major non-government organizations’ (NGOs) websites on July 18, 2016: WHO, Oxfam International, Save the Children, Community Health Workers Central (CHW Central), UNAIDS, and Target TB, UK. The search terms used are summarized in Table 1.

**Table 1:** Search strategy

Database	Search terms in title or abstract	No. of papers retrieved
PubMed	(healthcare workers OR healthcare providers OR healthcare professionals OR healthcare staff OR healthcare practitioners OR health workers OR health providers OR health professionals OR health staff OR health practitioners OR health-care workers OR health-care providers OR health-care professionals OR health-care staff OR health-care practitioners)	131,755
	AND (training OR continuing professional development OR continuing medical education)	16,588
	AND (evaluat* OR assess*)	7518
	AND (tuberculosis OR TB OR HIV OR malaria OR AIDS)	707
	Limit to articles published from January 1, 2000, to April 28, 2016	525
EMBASE	(healthcare workers OR healthcare providers OR healthcare professionals OR healthcare staff OR healthcare practitioners OR health workers OR health providers OR health professionals OR health staff or health practitioners OR health-care workers OR health-care professionals OR health-care providers OR health-care practitioners OR health-care staff)	166,542
	AND (training OR continuing professional development OR continuing medical education)	21,847
	AND (evaluat* OR assess*)	10,544
	AND (malaria OR AIDS OR HIV OR tuberculosis OR TB)	927
	Limit to publication year from 2000 to 2016	806
Cochrane Library	(healthcare workers OR healthcare providers OR healthcare professionals OR healthcare staff OR healthcare practitioners OR health workers OR health providers OR health professionals OR health staff or health practitioners OR health-care workers OR health-care professionals OR health-care providers OR health-care practitioners OR health-care staff)	21,999
	AND (training OR continuing professional development OR continuing medical education)	4984
	AND (evaluat* OR asses*)	3837
	AND (malaria OR AIDS OR HIV OR tuberculosis OR TB)	314
	Limit to publication year from 2000 to 2016	249

### Stage 3: study selection

All citations were imported into EndNote X7 and duplicate citations were removed manually. A two-stage screening process for eligibility was conducted. Articles were eligible for inclusion if the studies met the inclusion and exclusion criteria (Table 2). In the first stage of screening, two researchers independently reviewed titles and abstract of the citations. Results from both researchers were compared, and titles for which an abstract was not available or for which either of the reviewers’ suggested inclusion were put forward for subsequent full-text review as part of the second stage of eligibility screening. If the studies did not meet the eligibility criteria, they were excluded at this stage. Articles that could not be obtained through online databases and library searches at the National University of Singapore and London School of Hygiene and Tropical Medicine were also excluded from final analysis.

**Table 2:** Inclusion and exclusion criteria

Inclusion criteria	• Study describes evaluations of HIV, malaria, or TB HCP post-graduate training programs • Study contains descriptions of the training program, methods used to evaluate the program and outcomes assessed in the evaluation. • Study was published after January 1, 2000. • Geographic areas of studies are not restricted. • Only published articles will be included.
Exclusion criteria	• Literature reviews with no primary data collection • Study describes framework or methodology proposed for training evaluation without primary data collection and analysis.

### Stage 4: extracting and analyzing the data

We extracted relevant information from articles included in the final analysis using a pre-designed standardized excel sheet. Table 3 summarizes data extracted and definitions used for categorizing data. For each study, we categorized the training outcomes evaluated into four levels (reaction, learning, behavior, and results) based on the Kirkpatrick model.

**Table 3:** Definitions of extracted data

Data extracted	Definition
Year of publication	Year in which the study was published
Study location	Country in which the study took place
Disease area	The disease area that the training program aimed to target (HIV, malaria, or tuberculosis).
Evaluation methods	
Pre- and post-training tests	Trainees were given tests on their knowledge acquisition before and after training sessions. Scores of both tests were compared.
Quantitative survey of trainees	Trainees’ feedback, demographic information, or other key information used for evaluation were collected using questionnaires filled out by either trainees or evaluators via one-to-one interviews. Data was analyzed using quantitative methods.
Qualitative interviews	Trainees were interviewed one-to-one by evaluators after training. Information was collected through in-depth or semi-structured interviews. Data was analyzed using qualitative methods.
Review patient records	Patient records were extracted and patient level outcomes were compared before and after the training program or between intervention and control groups. Data sources included medical records at health facilities, patient cards, or local surveillance data.
Patient exit survey	After training programs, patients were surveyed by evaluators after consultations with trainees. A standardized questionnaire was used to record the services received by patients, drugs prescribed, or whether they were satisfied with the consultations.
Observation	Trainees’ on-the-job performance was directly observed at their work place and assessed by evaluators or their supervisors.
Standardized patient	Standardized patients refer to people trained to accurately portray a specific medical condition. In this method, trainees’ performance was evaluated during clinical encounters without the presence of evaluators.
Focus group discussion	Trainees were gathered in groups after training programs to discuss their experiences, feedback, and reflections on the training programs. The discussion was usually guided by a facilitator.
Cost-effective analysis	The cost of the training program was calculated and compared with the outcomes of the program.
Outcomes evaluated	
Reaction	How trainees react to the training and their perceived value of the training
Learning	To what degree trainees acquire intended knowledge, skills, and attitudes based on participation in the learning event
Behavior	To what degree trainees apply what they learned during training sessions on their job
Results	The downstream organizational outcomes/impacts that occur as a result of the training

### Stage 5: collating, summarizing, and reporting the results

Guided by the research question, we summarized the results on characteristics of the included studies using descriptive statistics.

## Results

### Summary of included studies

**Fig. 1:**
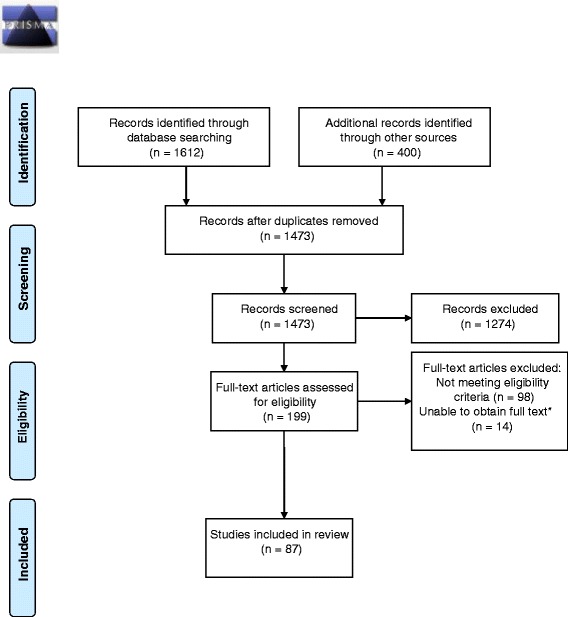
Flowchart of literature search and screening. *Full text not available through National University of Singapore and London School of Hygiene and Tropical Medicine

We retrieved a total of 1612 citations from the three databases and 400 from the gray literature search. After removing duplicates, we screened titles and abstracts of 1473 unique publications, of which 199 went through to the full-text assessment and 87 met our inclusion criteria for inclusion in the analysis (Fig. 1).

We found that the number of published articles on HCP training evaluation has increased, particularly after 2006 (Table 4). In terms of geographic distribution of studies, most (*n* = 57, 66%) took place in African countries. Compared to the number of studies in Africa, only 16 (18%) evaluation studies took place in Asian countries, and even fewer were conducted in North America (*n* = 10, 11%), Europe (*n* = 2, 2%), and South America (*n* = 2, 2%). The majority of training evaluations—44 studies (51%)—were based on HCPs providing HIV-related health services. Fewer studies were focused on HCPs providing malaria (*n* = 28, 32%) and TB (*n* = 23, 26%) related health services.

**Table 4:** Summary of included studies

Characteristic	Number of studies (*n* = 87)	Percentage (%)
Publication year		
2000–2002	2	2
2003–2005	4	5
2006–2008	12	14
2009–2011	25	29
2012–2014	31	36
2015–2016	13	15
Study location		
Africa	57	66
Asia	16	18
Europe	2	2
North America	10	11
South America	2	2
Disease area		
HIV	44	51
TB	23	26
Malaria	28	32

### Evaluation methods used in the studies

**Fig. 2:**
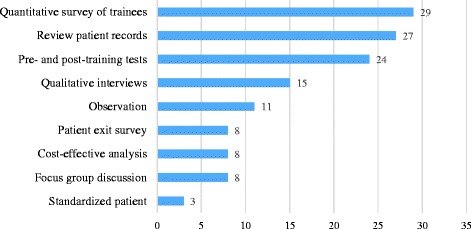
Number of studies that apply each method

A wide range of training evaluation methods was used. As shown in Fig. 2, the most common method was a quantitative survey of trainees (*n* = 29, 33%). Methods such as reviewing patient records (*n* = 27, 31%) to assess diagnostic and treatment outcomes after HCPs attended training session and pre- and post-training tests (*n* = 24, 28%) were also applied frequently. In contrast, only three studies (3%) evaluated on-the-job behavior change of trainees using standardized patients.

### Outcomes assessed in the included studies

In terms of the outcomes evaluated among the included studies, more than half of the included studies (*n* = 44, 51%) evaluated knowledge acquisition (learning) of trainees after training sessions and 38 (44%) studies evaluated downstream results of the training programs. Fewer studies (*n* = 16, 18%) assessed whether trainees liked the program or whether the program was considered useful for trainees (reaction), and 30 (34%) measured the behavior change of trainees after they finished the training and returned to their jobs.

As summarized in Table 5, among the 16 studies evaluating trainees’ reaction, more than half (*n* = 9, 56%) conducted a quantitative survey with trainees after training and only two (13%) used pre- and post-training tests to investigate whether trainees liked the training programs or felt the programs were useful to them. In terms of the learning level, the most commonly used method was pre- and post-training tests (*n* = 23, 52%). Qualitative methods such as interviews (*n* = 4, 9%) or focus group discussion (*n* = 4, 9%) were also used, albeit less frequently, when assessing knowledge gain of trainees. Observation was used commonly when assessing behavior change of trainees (11, 37%). Quantitative survey with trainees (*n* = 9, 30%) and qualitative interviews (*n* = 7, 23%) were also used to record self-reported behavior change of trainees after training programs. Additionally, three studies (10%) used standardized patients when evaluating on-the-job performance of trainees after training sessions. Finally, in terms of results on an organizational level, review of patient records (*n* = 26, 68%) was the most commonly used method. Patient exit survey (*n* = 8, 21%) was also used to assess patients’ experiences and satisfaction with the services provided by trainees. Even though cost of the training programs was an important indicator for program managers and policy makers, only eight studies (21%) conducted evaluations on the cost of the programs.

**Table 5:** Common evaluation methods for each level of the Kirkpatrick model

Level of evaluation	Common methods used	Number of studies^a^	Percentage (%)	References^a^
Reaction (*n* = 16)	Quantitative survey of trainees	9	56	[23, 32–39]
	Qualitative interview	5	31	[40–44]
	Focus group discussion	4	25	[36, 38, 42, 45]
	Pre- and post-training tests	2	13	[46, 47]
Learning (*n* = 44)	Pre- and post-training tests	23	52	[27, 33–35, 37, 43, 46–62]
	Quantitative survey	16	36	[23, 45, 63–76]
	Qualitative interview	4	9	[44, 69, 77, 78]
	Focus group discussion	4	9	[51, 56, 78, 79]
Behavior (*n* = 30)	Observation	11	37	[32, 33, 39, 42, 57, 63, 80–84]
	Quantitative survey of trainees	9	30	[23, 45, 62, 64, 67, 70, 72, 77, 85]
	Qualitative interview	7	23	[44, 69, 77, 78, 86–88]
	Standardized patient	3	10	[56, 89, 90]
	Review patient records	1	3	[85]
	Pre- and post-training tests	1	3	[61]
Results (*n* = 38)	Review patient records	26	68	[27, 28, 42, 57, 58, 66, 78, 80, 87, 91–107]
	Patient exit survey	8	21	[35, 63, 67, 108–112]
	Cost-effective analysis	8	21	[33, 35, 42, 57, 92, 97, 108, 109]
	Quantitative survey of trainees	2	5	[113, 114]
	Qualitative interview	1	3	[78]

^a^Articles may be double entered in this column

## Discussion

Our paper provides the first synthesis of methods applied and outcomes assessed in studies evaluating HCP training for HIV, malaria, and TB service delivery. Overall, we found a fairly limited number of published evaluation studies of HCP training programs, especially in light of the number of training programs implemented since 2000. Among the 87 training evaluation studies identified, the most commonly applied assessment methods were quantitative surveys and reviews of patient records and the most commonly assessed outcomes were learning and downstream results. Specific gaps in the literature identified were evaluations of TB- and malaria-related HCP training, evaluations conducted in Asian countries with high disease burden, and studies providing objective information on behavior change of trainees or costs of training.

While a substantial proportion (51%) of studies assessed “learning” of trainees, we found that most used pre- and post-training tests. This is likely because tests can be conducted fairly easily after training sessions without further follow-up. However, knowledge assessments using pre- and post-training tests have limitations with many experts in psychology and education stressing that knowledge acquisition is a dynamic process that may not be captured through a simple paper-based assessment [17]. Tests at the end of the training sessions are best suited for testing retention of factual knowledge [21], but for most HCP training programs, improvements in service quality are as important as retained knowledge. Therefore, assessment of behavior change of HCPs after attending training programs is critical in determining whether the objectives of the training interventions have been achieved; our findings revealed that only 30 studies across all three diseases assessed behavior change. Furthermore, behavior change was most commonly assessed through direct observation of trainees’ on-the-job performance by evaluators, a method which would result in a high risk of bias because trainees’ behavior would likely be altered when evaluators observed their performance during consultations [22]. Surveys and qualitative interviews asking participants if they have applied newly acquired skills were other methods, also subject to bias, commonly used in assessing behavior change of trainees [23]. While self-reporting behavior may vary by cultural context, there is a risk that trainees may not be willing to reveal that they are not using the skills learned at the training sessions to evaluators, who are often involved in conducting the original training.

An alternative method for assessing the behavior of HCP is through the use of standardized patients, which refers to people trained to accurately portray a specific medical condition [24]. This method—used in some medical schools for evaluating clinical performance—provides a structured way for evaluators to capture trainees’ clinical competence and communication skills. Compared to direct observation, it minimizes bias because trainees do not know when a clinical encounter with standardized patients will occur [24, 25]. We found that this assessment method is rarely used in the evaluation of HCP training programs, possibly because it is resource and time consuming to find and train standardized patients.

In addition to assessing learning or knowledge gain, downstream results were also widely evaluated in HCP training programs. As part of these evaluations, researchers typically compared patient-level outcomes before and after training programs or between intervention and control groups by reviewing clinical records of patients who were treated by trainees participated in the training programs. For example, in TB control programs, indicators from standard guidelines, such as treatment success rate and case detection, were used as outcome indicators in evaluation of TB HCP training programs [26]. Likewise, in HIV-related training programs, indicators such as HIV testing rate and proportion of patients with undetectable viral loads were used as outcome indicators in the training evaluation [27, 28]. However, changes in downstream organizational results, such as improved case detection or treatment success rate used in TB training programs and proportion with undetectable viral loads in HIV training programs, cannot be simply attributed to HCP training programs using, for example, a before-after evaluation approach, because training programs are often embedded within a broader national control strategy with other prevention and control activities ongoing in parallel. Downstream health outcomes are challenging to assess as they are multifactorial and complex. Other factors, such as improved supply of medical equipment or enhanced healthcare infrastructure, may also contribute to better patient outcomes. These evaluations also tend to rely on routine patient records which may vary in accuracy and completeness.

When considering impact on the wider organization or disease control program, costs of training were not widely assessed. Only eight studies assessed the cost of the training programs, even though cost is an important indicator to policymakers in making decisions on resource allocation [29].

In this scoping review, the goal-based Kirkpatrick model was used in categorizing evaluation outcomes of included studies. Even though developed in the 1960s, the Kirkpatrick model is still the most commonly used evaluation framework and formed the foundation for other goal-based evaluation frameworks developed subsequently [12–18]. For example, in the Phillips model, a fifth level, return on investment (ROI), was added to the classic four-level Kirkpatrick model to assess the cost-benefit of the training [16]. Another example is the Hamblin’s five-level model, in which the result level in the Kirkpatrick model was split into two: organization and ultimate value [30]. The organization level assesses the impact on organization from the behavior changes of trainees, and the ultimate value measures the financial effects of the training program on the organization and the economy [30]. Apart from the goal-based models for training evaluation, which intend to determine whether the goals set before the start of the training were achieved, system-based models that focus on the assessment of the context and the process of the training program were also developed to guide the evaluation [31]. However, compared to goal-based models, very few system-based models provide detailed description of process involved and outcomes needed to be assessed in each step of the evaluation, which makes them less popular among evaluators [31].

In order to conduct a broad search of gray and published literature, we included three electronic databases, Google scholar, and six NGO websites and did not set language limits to exclude studies published in languages other than English. However, we recognize that we may have missed some HCP training evaluations if the studies were not published or accessible online. Additionally, since we intended to include published peer-reviewed evaluation studies, we did not analyze studies published as conference abstracts or presented as posters at conference in this review. As recognized in other scoping reviews as well, the quality of the included studies was not assessed, because the primary aim was to summarize the range of existing literature in terms of their volume, nature, and characteristics [21]. The lack of rigorous HCP training evaluation studies in current literature may reflect the limited knowledge, experience, and budget available to program managers or researchers in LMICs to conduct training evaluations. A limitation of our study is that we did not analyze qualitative information about challenges with conducting training evaluations mentioned in the studies identified; a further systematic review and analysis of the limitations mentioned in existing training evaluation studies or in interviews with program managers could be conducted in future to investigate barriers and difficulties encountered by evaluators when conducting training evaluations, particularly in LMICs. Such studies would be useful to identify strategies to increase the evidence base in this area. In addition, future studies on development of standardized training evaluation frameworks or methods would also be helpful to minimize biases in assessment, improve accountability of evaluation results, and make HCP training evaluation more relevant to policymakers.

## Conclusions

Evaluations are critical to determine the effectiveness of HCP training in order to inform decisions on future investments. However, our study found limited evidence from robust evaluations conducted since 2000, even though the number of training interventions has increased over this period to address the shortage of HRH for HIV, malaria, and TB control globally. Specifically, we found a limited number of evaluation studies on TB- and malaria-related HCP training and very few studies assessing behavior change of trainees or costs of training. More evidence from well-designed HCP training evaluations is needed, and this may be facilitated by developments in frameworks and standardized methods to assess impacts of training.

## Abbreviations

HCP: Healthcare provider
HRH: Human resources for health
LMICs: Low- and middle-income countries
TB: Tuberculosis
The Global Fund: The Global Fund to Fight AIDS, TB and Malaria
